# Ambient fine particulate matter in Latin American cities: Levels, population exposure, and associated urban factors

**DOI:** 10.1016/j.scitotenv.2021.145035

**Published:** 2021-06-10

**Authors:** Nelson Gouveia, Josiah L. Kephart, Iryna Dronova, Leslie McClure, José Tapia Granados, Ricardo Morales Betancourt, Andrea Cortínez O'Ryan, José Luis Texcalac-Sangrador, Kevin Martinez-Folgar, Daniel Rodriguez, Ana V. Diez-Roux

**Affiliations:** a Department of Preventive Medicine, University of Sao Paulo Medical School, Sao Paulo, Brazil; b Urban Health Collaborative, Drexel Dornsife School of Public Health, Philadelphia, PA, USA; c Department of Landscape Architecture & Environmental Planning, College of Environmental Design, University of California Berkeley, Berkeley, CA, USA; d Department of Epidemiology and Biostatistics, Dornsife School of Public Health, Drexel University, Philadelphia, PA, USA; e Department of Politics, College of Arts & Sciences, Drexel University, Philadelphia, PA, USA; f Department of Civil and Environmental Engineering, Universidad de Los Andes, Bogotá, Colombia; g Pontificia Universidad Católica de Chile, Department of Public Health, School of Medicine, Chile; h Universidad de La Frontera, Department of Physical Education, Sports and Recreation, Chile; i Department of Environmental Health, Center for Population Health, National Institute of Public Health, Mexico; j Instituto de Nutrición de Centroamérica y Panamá (INCAP), Guatemala; k Department of City and Regional Planning and Institute for Transportation Studies, University of California, Berkeley, CA, USA

**Keywords:** Air pollution, Particulate matter, Built environment, City planning

## Abstract

**Background:**

Exposure to particulate matter (PM_2.5_) is a major risk factor for morbidity and mortality. Yet few studies have examined patterns of population exposure and investigated the predictors of PM_2.5_ across the rapidly growing cities in lower- and middle-income countries.

**Objectives:**

Characterize PM_2.5_ levels, describe patterns of population exposure, and investigate urban factors as predictors of PM_2.5_ levels.

**Methods:**

We used data from the Salud Urbana en America Latina/Urban Health in Latin America (SALURBAL) study, a multi-country assessment of the determinants of urban health in Latin America, to characterize PM_2.5_ levels in 366 cities comprising over 100,000 residents using satellite-derived estimates. Factors related to urban form and transportation were explored.

**Results:**

We found that about 172 million or 58% of the population studied lived in areas with air pollution levels above the defined WHO-AQG of 10 μg/m^3^ annual average. We also found that larger cities, cities with higher GDP, higher motorization rate and higher congestion tended to have higher PM_2.5_. In contrast cities with higher population density had lower levels of PM_2.5_. In addition, at the sub-city level, higher intersection density was associated with higher PM_2.5_ and more green space was associated with lower PM_2.5_. When all exposures were examined adjusted for each other, higher city per capita GDP and higher sub-city intersection density remained associated with higher PM_2.5_ levels, while higher city population density remained associated with lower levels. The presence of mass transit was also associated with lower PM_2.5_ after adjustment. The motorization rate also remained associated with PM_2.5_ and its inclusion attenuated the effect of population density.

**Discussion:**

These results show that PM_2.5_ exposures remain a major health risk in Latin American cities and suggest that urban planning and transportation policies could have a major impact on ambient levels.

## Highlights

• Cities in Latin America with higher GDP, motorization rate, and congestion have higher PM_2.5_.
• Cities in Latin America with higher population density and green space have lower levels of PM_2.5_.
• Intersection density and mass transit infrastructure also impact pollution levels.
• Urban planning and transportation policies may have a major impact on air pollution.

## Graphical abstract

**Figure:**
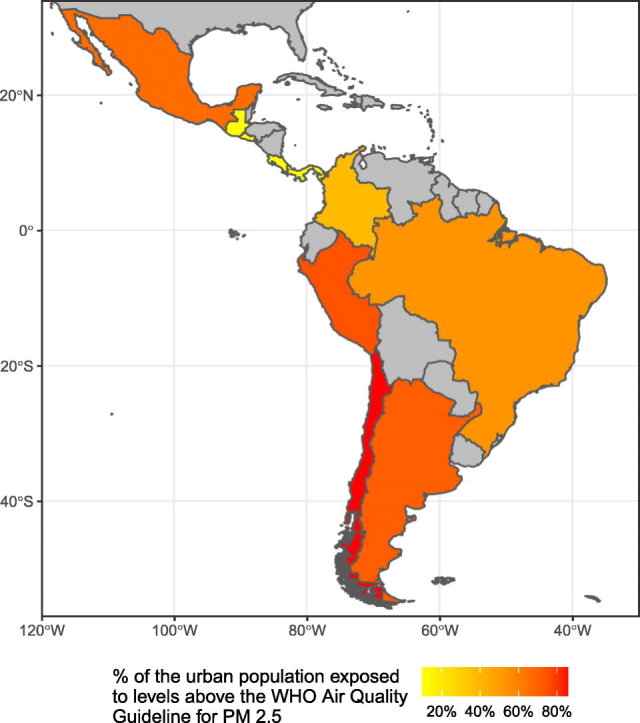

## 1 Introduction

Ambient air pollution is associated with a variety of mortality and morbidity outcomes ranging from cardiovascular and respiratory diseases to pregnancy and early life outcomes (Landrigan et al., 2018). It is also known that air pollution differentially affects young children and the elderly (Gouveia and Fletcher, 2000; Peled, 2011).

Recent estimates from the World Health Organization (WHO) indicating that nine out of 10 people in the world breathe air containing high levels of pollutants (WHO, 2018) reinforce the role of poor ambient air quality as a major threat to human health. Residents of urban areas are often at an especially high risk of exposure due to the concentration of mobile and stationary air pollution sources (e.g., traffic, industry, energy production) in and around cities. Urbanization has been increasing rapidly world-wide and it is estimated that by 2050 about 2/3 of the world's population will live in urban areas (UN DESA, 2018).

This growth of urban residents is predicted to be particularly pronounced in lower- and middle-income countries (LMIC) (Cohen, 2004). Therefore, it is especially important to identify ways in which cities can be designed, built and governed in order to reduce levels and exposure to health damaging air pollution. Furthermore, as the global climate continues to change, the well-established adverse interactions between heat and air pollution add urgency to the need to understand and respond to these air pollution challenges.

The Latin America and the Caribbean (LAC) region is one of the most urbanized areas of the world. Approximately 80% of the region's population resides in urban areas (World Bank, 2020) and 2/3 live in agglomerations of more than 20,000 inhabitants (ECLAC, 2018). Regulatory and control measures to improve ambient air quality in most countries in LAC have generally been insufficient (Riojas-Rodríguez et al., 2016). Hence, millions of urban residents in this region remain at high risk of adverse health effects from widespread exposure to particulate matter (PM) and other air pollutants. Besides the severe health consequences of exposure, there are also a range of economic implications: from an increased need for medical care, to lower productivity, and decreased quality of life (Hoek et al., 2013; Pope III et al., 2009; Shepherd et al., 2016; Graff Zivin and Neidell, 2012).

A WHO report estimated that over 110 million people were exposed to unhealthy levels of air pollution and approximately 58,000 deaths per year could be attributable to ambient air pollution in LAC (WHO, 2014). More recently, Riojas-Rodríguez et al. (2016) observed that the annual mean values of PM_10_ (particulate matter smaller than 10 μm in aerodynamic diameter) and PM_2.5_ (particulate matter smaller than 2.5 μm in aerodynamic diameter) in most measured sites in LAC were significantly higher than WHO Air Quality Guidelines (WHO-AQG), with less than 5% of cities (among 117 studied) complying with the guidelines (WHO, 2006).

Although informative, prior attempts to characterize exposure in urban areas of LAC relied only on information provided by the limited network of air quality monitoring stations available. Riojas-Rodríguez et al. (2016) note that only 117 cities in 33 LAC countries had official information on ground-level urban air pollutants. In addition, although other studies have focused on cities in China, Germany, and the US, to our knowledge, no study has examined the association of air pollution with urban characteristics such as city size, growth rate and built environment features in LAC (Wu et al., 2015; McCarty and Kaza, 2015; Liu et al., 2018). These characteristics of the urban environment have been shown to be particularly helpful in explaining pollution (Ye et al., 2016), particularly in contexts where air quality data are limited or non-existent (Weber et al., 2014). Thus, understanding the relation between policy-amenable features of cities and air pollution levels in urban areas can be critical to the development and promotion of urban policies to protect both population health and the environment.

We used data from the Salud Urbana en America Latina/Urban Health in Latin America (SALURBAL) study (Diez Roux et al., 2019), a multi-country assessment of the determinants of urban health in Latin America, to characterize PM_2.5_ levels in 366 cities with over 100,000 residents in the region using satellite-derived estimates. We described the patterns of population exposure, and compared current levels to the WHO-AQG. In addition, we investigated several policy-relevant city and sub-city urban factors as predictors of PM_2.5_ levels. We hypothesized that denser cities, less fragmented cities, greener cities, less congested cities, and those with better public transportation and higher gas prices would have lower levels of PM_2.5_.

## 2 Methods

SALURBAL encompasses all cities with population over 100,000 in 11 countries in Central and South America: Argentina, Brazil, Chile, Colombia, Costa Rica, El Salvador, Guatemala, Mexico, Nicaragua, Peru, and Panama (Diez Roux et al., 2019). SALURBAL identified cities using various administrative and quantitative criteria and used a two-level tiered system to define cities and their smaller subunits using census hierarchies (Quistberg et al., 2019). In these analyses, cities were defined as clusters of one or more administrative units (herein referred to as sub-city units) that together captured the visually apparent urban extent of the identified urban agglomerations (Quistberg et al., 2019). The sub-city units were the smallest administrative units for which vital statistic data were routinely available (e.g. *departamentos*, *municipios*, *comunas* depending on the country).

Together, the SALURBAL cities in the 11 countries include approximately 300 million residents, representing more than half of the total population in Latin America, and comprise some of the largest cities in the world, such as Sao Paulo, Mexico City, Buenos Aires, Lima, and Rio de Janeiro. Nicaragua was not included in these analyses because of missing data on several key variables.

### 2.1 Air pollution data

Air pollution data were obtained from the Atmospheric Composition Analysis Group of the Dalhousie University (surface PM_2.5_ global estimates available from: http://fizz.phys.dal.ca/~atmos/martin/?page_id=140). Ground-level fine particulate matter (PM_2.5_) was estimated using multiple satellite-based aerosol optical depth datasets combined with a chemical transport model, and subsequently calibrated to global ground-based observations using geographically weighted regression (Van Donkelaar et al., 2016). Data were available as annual means (μg/m^3^) in a gridded format with each grid cell representing 0.01 × 0.01 degrees, equivalent to 1.1 km × 1.1 km at the equator. We converted the value of each grid cell into points assigned to the geometric center of each cell (centroids). We calculated annual mean PM_2.5_ value in the year 2015 for each sub-city unit by averaging the values of each centroid contained within the unit boundary. We used all composition PM_2.5_ to better reflect the exposure of the population.

### 2.2 City and sub-city factors

We examined several city and sub-city factors related to urban form and transportation based on our hypotheses. We also included the gross domestic product (GDP), population size and city growth as they may also affect PM_2.5_. We determined whether the factor should be included at the city or sub-city level based on (1) theoretical understanding regarding at what level the construct is defined (for example, features such as city growth and urban fragmentation are more meaningful for the city as a whole than for smaller areas within a city), and (2) whether significant and meaningful within-city variation was likely (e.g., greenness may vary substantially within a city and is meaningfully described as a sub-city level construct).

Urban form factors at the city level included population density (population divided by built-up area), and a measure of urban fragmentation (patch density: the number of contiguous patches of urban development per km^2^ of area). At the sub-city level, we examined intersection density (street node density of the set of nodes with more than one street emanating from them, which indicates connectedness and walkability (McCarty and Kaza, 2015) per km^2^ of area) and area median greenness measured by the normalized difference vegetation index (NDVI). NDVI was calculated using MODIS satellite-based observations from the MODIS vegetation product, MOD13Q1.006 for 2015 at a 250 m spatial resolution. We computed the maximum NDVI value for the year at 250 m resolution to present the ‘greenest’ condition of each grid cell, then calculated the median across grid cells contained within each sub-city unit.

Transportation at the city level was characterized based on the presence of mass transit infrastructure such as subway or Bus Rapid Transit (defined as present or absent) and an indicator of gas cost (the cost of 100 l/monthly minimum wage). We also calculated a travel delay index which measures the average congested travel time from a set of 25 random origin-destination points relative to the uncongested time using the street network. This indicator was constructed based on the largest built-up urban cluster within each administratively defined city, as this spatial definition is most relevant to characterizing congestion. The urban clusters were identified using the Global Urban Footprint Dataset as described in Quistberg et al. (2019).

We also gathered data on motorization rate in 2015 for a small subsample of cities for which these data were available. The motorization rate (per 1000 population) is the total number of registered motor vehicles in 2015 which includes light and heavy-duty vehicles.

Indicators of city size and growth included total city population in 2015 and population growth (%) between 2010 and 2015. We also examined the GDP per capita (computed as purchasing power parities in constant 2011 international USD) of each city in 2015 using estimates from the first subnational administrative level (typically equivalent to departments or states) as described by Kummu et al. (2018).

### 2.3 Analysis

PM_2.5_ levels were analyzed at the sub-city level in order to capture any variations within cities composed of multiple sub-city units. Descriptive statistics, boxplots, and variance components from multilevel models were used to describe variability in PM_2.5_ levels among countries, cities and sub-cities. Because of the relatively smaller number of cities within Costa Rica, Guatemala, El Salvador, and Panama, we pooled data from these countries into one region of Central America for the analysis. We also described the population exposed to levels of PM_2.5_ above the WHO-AQG.

We used linear mixed models with random intercepts at the country and city level and an unstructured covariance structure to examine the association of selected sub-city and city level factors (the predictors) with mean PM_2.5_ for sub-city units, and to estimate variance at each level (within cities, between cities within countries, and between countries). Each predictor was first examined separately. We adjusted the measure of urban fragmentation (patch density) by the percentage of the administrative area that is built-up (% built-up), in order to account for differences in the amount of development in each area and thus, correctly interpret the coefficient of patch density as an indicator of fragmentation. In a second phase we included all predictors in a single full model. To facilitate comparisons, all city and sub-city factors were standardized to standard deviation (SD) units.

## 3 Results

Of the 371 cities in SALURBAL, five were excluded because they were in Nicaragua. A total of 366 cities and 1425 sub-cities were included in the analyses describing the distribution of the population above the WHO-AQG. For the multilevel analysis, we excluded cities missing city and sub-city level predictors; thus, the final sample included 343 cities and 1310 sub-cities in 10 Latin American countries. Forty-eight percent of cities were composed of only one sub-city unit. The median number of sub-cities within cities with more than one unit for the full sample was four but ranged from two in Argentina to 18 in the pooled Central America countries (Table 1).

**Table 1:** Characteristics of cities and sub-cities included in analyses.

	Argentina	Brazil	Central America	Chile	Colombia	Mexico	Peru
City level characteristics (median, 25th and 75th %tiles)^a^							
Number of cities	32	145	10	21	35	77	23
Population in 100,000s, 2015	3.3 (2.0, 5.8)	2.6 (1.6, 5.6)	2.8 (2.4, 19.4)	2.4 (1.7, 3.7)	3.7 (1.8, 5.9)	3.4 (2.1, 7.4)	3.0 (2.0, 4.7)
Population growth %, 2010 to 2015	6.0 (5.1, 7.1)	5.4 (3.7, 7.4)	7.6 (5.2, 9)	5.7 (4.6, 9.3)	6.2 (3.2, 8.6)	5.1 (1.2, 7.5)	6.4 (5.6, 8.2)
GDP per capita (purchasing power parity)	19.6 (11.6, 22.4)	14.8 (8.6, 21.0)	10.8 (7.8, 16.4)	17.7 (13.0, 26.6)	11.8 (8.6, 13.8)	13.3 (10.9, 16.2)	8.0 (6.7, 12.8)
Presence of mass transit infrastructure %	9	18	30	14	20	9	4
Gas affordability: (cost of 100 l/monthly minimum wage)	2.0 (1.9, 2.1)	3.7 (3.5, 3.9)	2.7 (1.6, 3.7)	2.6 (2.6, 2.7)	2.6 (2.6, 2.6)	6.7 (6.7, 6.8)	3.4 (3.3, 3.6)
Urban fragmentation (patches/km^2^)	0.1 (0.1, 0.3)	0.3 (0.1, 0.6)	0.7 (0.6, 0.9)	0.3 (0.1, 0.5)	0.2 (0.1, 0.5)	0.4 (0.2, 0.6)	0.1 (0.1, 0.3)
Population density (pop. in 1000s/km^2^ of built-up area)	5.3 (4.8, 6.1)	6.1 (5.1, 7.8)	9.5 (7.5, 12.8)	6.8 (6.2, 8.6)	15.3 (13.1, 18.9)	6.3 (5.5, 7.4)	11.8 (9.7, 13.5)
Travel delay index^c^	0.12 (0.09, 0.15)	0.12 (0.08, 0.16)	0.24 (0.19, 0.68)	0.31 (0.27, 0.33)	0.36 (0.26, 0.47)	0.18 (0.13, 0.24)	0.22 (0.14, 0.29)
Motorization rate^d^	–	540 (404, 648)	–	251 (235, 270)	–	372 (310, 450)	–

Sub-city level characteristics (median, 25th and 75th %tiles)^a^							
Number of sub-cities	108	406	153	81	83	311	168
Number of sub-cities in cities with >1 sub-cities, median (10th, 90th %tile)	2 (2, 7)	4 (2, 10)	18 (9, 39)	3 (2, 10)	3 (2, 6)	3 (2, 12)	5 (2, 11)
Intersection density (per km^2^)	13.1 (2.4, 73.3)	8.2 (3.9, 19.9)	12.8 (3.1, 46.4)	23.5 (4.5, 100.7)	5.1 (1.9, 10.6)	5.8 (2.6, 15.6)	14.2 (3.8, 78.3)
Greenness index	0.63 (0.36, 0.78)	0.81 (0.77, 0.85)	0.85 (0.73, 0.88)	0.61 (0.26, 0.74)	0.81 (0.77, 0.84)	0.73 (0.66, 0.83)	0.33 (0.19, 0.59)
Annual mean PM_2.5_^b^ in μg/m^3^ in 2015	12.1 (9.0, 15.5)	9.7 (6.7, 13.9)	7.8 (5.1, 9.0)	20.1 (12.9, 31.0)	8.3 (7.1, 10.4)	14.5 (10.1, 18.4)	14.6 (7.6, 22.5)

a Considering 343 cities and 1310 sub-cities in 10 Latin American countries after excluding cities with missing covariates.

b Full sample of 366 cities and 1425 sub-cities.

c Measured for the built-up urban core of the city.

d Available only for 241 cities in Brazil, Chile and Mexico.

Descriptive characteristics of cities and sub-cities are displayed in Table 1. Overall, the median population size and growth among cities is similar across countries. On the other hand, urban form and transport characteristics such as population density and travel delay varied considerably across countries, with lower population density in Argentina and higher in Colombia, and travel delay lowest in Argentina and Brazil and highest in Colombia. The level of fragmentation and the percentage of built-up area also varied among cities and countries. The distribution of the greenness index (NDVI) varied from a median of 0.33 in Peru to 0.85 in Central America. For a smaller subsample of cities in three countries for which data on motorization rates were available, we observed that Brazil had the highest median, followed by Mexico and Chile.

**Fig. 1:**
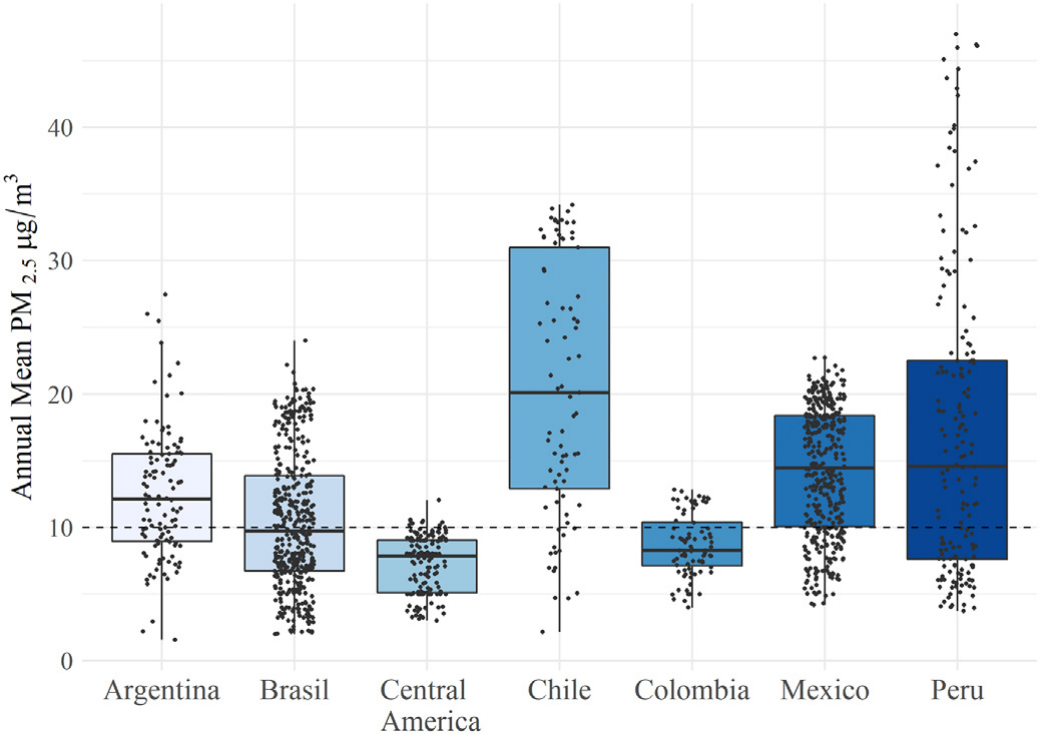
Annual mean PM_2.5_ in 1425 sub-cities from 366 cities in Latin America, with WHO annual guideline for PM_2.5_ (10 μg/m^3^).

Annual mean levels of PM_2.5_ varied between and within countries (Table 1 and Fig. 1). Of all the variation across sub-city units, only 9% was between countries, revealing substantial within-country variability. In fact, PM_2.5_ varied substantially both within cities and between cities within a country. Of the total variability in PM_2.5_ levels, 34% was between sub-city units within a city and 57% between cities within a country. Similar results were obtained when analyses were restricted to cities with more than one sub-city unit.

In 2015, 55% of the sub-cities (*n* = 783) had annual mean concentrations of PM_2.5_ above the defined WHO-AQG of 10 μ/m^3^ (WHO, 2006). The proportion of sub-cities above the WHO-AQG varied substantially among the countries analyzed, ranging from a high of 83% of the sub-cities in Chile to around 6% in Central America (Fig. 1).

Of the 295 million residents in our sample of 366 Latin American cities, approximately 171 million (58% of total) lived in areas (sub-city units) with PM_2.5_ concentrations that exceed WHO guidelines. Among this population, around 12 million were children under five years of age and 14 million were elderly people (over age 65), age groups traditionally considered at higher risk of health effects due to air pollution exposure (Table 2). The proportion of the population living in sub-cities where levels of PM_2.5_ exceeded the WHO-AQG varied markedly among the countries, ranging from 86% in Chile to around 10% in Central American countries (Table 2).

**Table 2:** Percentage of residents living in sub-cities with levels of PM_2.5_ above the WHO Guideline annual mean concentration of 10 μgm^3^_,_ by age (in parenthesis population above WHO-AQG in each category).

Country	Age < 5	Age ≥ 65	All ages
Argentina	71% (1,812,862)	74% (2,366,978)	71% (21,227,417)
Brazil	50% (4,030,336)	60% (5,374,538)	53% (62,236,144)
Central America	10% (95,853)	10% (79,564)	10% (1,139,304)
Chile	85% (744,906)	86% (1,107,322)	86% (10,968,452)
Colombia	38% (875,061)	36% (793,365)	38% (10,965,939)
Mexico	65% (3,769,308)	70% (3,471,367)	67% (51,444,741)
Peru	72% (1,052,541)	79% (953,185)	74% (13,160,574)
Total	56% (12,380,868)	62% (14,146,319)	58% (171,142,571)

A closer look at the 10 largest cities in our sample (Mexico City, Sao Paulo, Buenos Aires, Rio de Janeiro, Lima, Bogota, Santiago, Guadalajara, Monterrey, and Porto Alegre) showed that 95% of the population in these megacities live in areas with PM_2,5_ greater than WHO-AQG. These 10 cities alone host more than 100 million people. In these 10 cities there were over seven million children under five and nearly nine million elderly adults over 65 living in areas with concentrations of PM_2.5_ above 10 μg/m^3^.

When each predictor was examined separately, several city and sub-city characteristics were significantly associated with annual PM_2.5_ levels (Table 3, univariable model). City GDP per capita and city population size were positively associated with PM_2.5_, i.e., the larger the population and the higher the GDP, the higher the annual mean of air pollution. On the other hand, higher population density was significantly associated with lower levels of air pollution. Higher travel delay was also associated with higher levels of air pollution. Sub-cities with higher intersection density had higher levels of PM_2.5_, and sub-cities with higher greenness had lower levels of PM_2.5_. City population growth and gas cost were inversely associated with PM_2.5,_ but the associations were not statistically significant at 95% confidence level. The level of urban fragmentation and the presence of mass transit infrastructure both showed positive associations with PM_2.5_, but those were also not statistically significant. In the subset of 241 cities with data on motorization, a higher motorization rate was associated with higher PM_2.5_ (mean difference in PM_2.5_ 1.55 95% CL 0.93, 2.18).

**Table 3:** Mean differences in annual mean PM_2.5_ μg/m^3^ concentrations at the sub-city level associated with a 1 SD higher value of city and sub-city-level characteristics.

	Univariable	Full model	Full model with motorization rate^c^
	Estimate (95% CI)	Estimate (95% CI)	Estimate (95% CI)
City factors			
GDP per capita	**1.00 (0.52, 1.47)**	**0.87 (0.43, 1.32)**	**0.65 (0.22, 1.09)**
Population	**2.57 (1.49, 3.65)**	0.01 (−1.54, 1.57)	−0.71 (−2.60, 1.18)
Population growth %, 2010 to 2015	−0.13 (−0.55, 0.30)	−0.29 (−0.66, 0.09)	−0.06 (−0.45, 0.32)
Mass transit infrastructure^a^	1.17 (−0.19, 2.53)	**−1.91 (−3.39, −0.42)**	**−1.87 (−3.40, −0.34)**
Gas cost	−0.17 (−1.68, 1.33)	−0.09 (−1.74, 1.56)	−1.75 (−4.36, 0.86)
Patch density^b^	0.47 (−0.31, 1.25)	0.64 (−0.18, 1.46)	0.67 (−0.21, 1.56)
Population density	**−0.71 (−1.41, −0.01)**	**−0.90 (−1.60, −0.20)**	−0.84 (−1.87, 0.18)
Travel delay index	**1.05 (0.13, 1.97)**	0.26 (−0.70, 1.22)	−0.62 (−2.09, 0.84)
Motorization rate	**1.55 (0.93, 2.18)**		**0.78 (0.12, 1.43)**

Sub-city factors			
Intersection density	**1.92 (1.70, 2.14)**	**1.91 (1.65, 2.17)**	**1.96 (1.67, 2.25)**
Greenness	**−1.39 (−1.72, −1.07)**	0.13 (−0.23, 0.49)	0.06 (−0.34, 0.46)

Note: figures in bold are statistically significant (p < 0.05).

a Binary presence or absence of mass transit infrastructure

b Measure of urban fragmentation that is additionally adjusted for z-standardized % built-up area.

c Based on subsample with 241 cities.

When all exposures were examined adjusted for each other (Table 3, full model), city per capita GDP and sub-city intersection density remained associated with PM_2.5_ levels, while population density was inversely associated with levels of air pollution. Also, the presence of mass transit infrastructure was associated with lower PM_2.5_. When motorization rate was added to the full model, other estimates remained similar, although the association with population density was attenuated (mean difference −1.05 95% CL −2.11, 0.02).

## 4 Discussion

To the best of our knowledge, this is the first study to examine patterns of population exposure and to assess urban factors associated with PM_2.5_ concentrations in a large sample of Latin American cities. We found that most cities in Latin America have levels of fine particle air pollution that exceed the recommended guidelines from the WHO and that these levels are associated with city and sub-city characteristics of the built environment.

On average, 55% of the sub-cities studied had levels of PM_2.5_ above WHO-AQG, with large variations among countries. This translates to about 172 million or 58% of the population studied living in areas with air pollution levels above the defined WHO-AQG of 10 μg/m^3^ annual average. In Chile, the proportion of the population living in cities or sub-cities that are above the guideline was as high as 86%, while in Central American countries, this proportion is only 10%. It is important to highlight that among the population exposed to high levels of particulate pollution, there are approximately 12 million children under age 5 and 14 million elderly people over age 65, populations most at risk of suffering the effects of this exposure. Children are particularly affected by air pollution because their lungs are still growing, their immune system is immature, they tend to spend more time outside, and they have higher breathing rates than adults (Kajekar, 2007; Dixon, 2002). The elderly may also be more affected by air pollution in part due to a larger burden of comorbidities (Gouveia and Fletcher, 2000).

We also found substantial spatial variability in levels of PM_2.5_ not only across cities but also across sub-city units with a third of the total variation observed within cities. This finding is consistent with studies that have shown that concentrations of air pollutants exhibit intra-city gradients and these can be attributed to factors like different building structures, traffic densities, wind speed and direction (Merbitz et al., 2012; Levy and Hanna, 2011). This finding suggests that city-wide averages (especially for large cities) hide important variation and may lead to underestimates of the population exposed to levels above the WHO-AQG.

A previous study (Riojas-Rodríguez et al., 2016) examined 117 Latin American cities encompassing approximately 146 million inhabitants. Using available air quality monitoring networks, they found that four out of 57 cities that measured PM_2.5_ were within the guidelines established by WHO. We expand this work by including a significantly larger number of cities and using satellite-derived PM measures. Only 84 of the 371 SALURBAL cities had monitoring stations for PM (either PM_10_ or PM_2.5_). The lack of extensive monitoring of air quality in Latin America makes it difficult to routinely assess the impact of the concentration of pollutants on health. It also hinders the population's perception and awareness of this important problem, and consequently, discourages efforts to ensure cleaner air for the population.

To overcome the relative scarcity of monitoring networks, information derived from satellites has been increasingly used to assess population exposure (Brauer et al., 2016; Shi et al., 2018). Satellite-based aerosol optical depth combined with chemical transport models and calibrated with ground level measurements enhance the spatial and temporal coverage of ground monitoring networks, provide a long-term view of air pollution levels, exposures and trends, and represent the most comprehensive PM_2.5_ data currently available at a global scale (Van Donkelaar et al., 2010). These data provide a picture of the average PM pollution that an individual living in a particular place would be exposed to on a typical day.

However, satellite derived air pollution estimates also have limitations. For example, they are not available at “real-time” as ground monitor readings since specific dates/times at which air pollution observations are available from the satellites are constrained by the specific times of satellite overpass. In addition, such data are typically of relatively coarse spatial resolution which makes them suitable for capturing broad-scale and city-level pollution patterns but limits the ability to relate these patterns to specific landscape composition and land cover/use, requiring other spatial datasets for such interpretations. Finally, they are limited to specific air pollutants and such data requires some degree of data management which makes their routine use difficult. Nevertheless, studies have shown a good comparability between satellite and monitoring data with no signs of systematic over or under-estimation of the ground-based monitored levels (Van Donkelaar et al., 2016; Duncan et al., 2014).

Using the rich data compiled by the SALURBAL project, we investigated how demographic, economic, built environment and transportation related features of cities (and smaller areas within cities) are related to levels of PM_2.5_. We found that larger cities, cities with higher GDP, and cities with higher congestion tended to have higher PM_2.5_. In contrast, cities with higher population density had lower levels of PM_2.5_. In addition, at the sub-city level, higher intersection density was associated with higher PM_2.5_ and more green space was associated with lower PM_2.5_. When all exposures were examined adjusted for each other, higher city per capita GDP and higher sub-city intersection density remained associated with higher PM_2.5_ levels, while higher city population density remained associated with lower PM_2.5_ levels. The presence of mass transit infrastructure was also associated with lower PM_2.5_ after adjustment.

Higher city GDP is likely a proxy for higher levels of industrialization and motorization. Higher GDP implies higher economic activity, which in turn means higher combustion of fossil fuels, the primary source of particulate air pollution including PM_2.5_. When compared to other contributing factors, there are indications that GDP per capita is the factor that offers the largest proportional contribution to PM_2.5_ concentrations (Zhao et al., 2019). In our analyses, higher GDP was associated with higher PM_2.5_ before and after adjustment for the other factors investigated.

As hypothesized, we found that denser cities had lower levels of PM_2.5_. This may be because compact, high-density cities, as opposed to sprawling urban developments, necessitate shorter average driving distances and thus less need for motorized transport, resulting in lower energy consumption, which in turn improves air quality. This relationship has been documented in an empirical study in 17 cities in Korea (Cho and Choi, 2014) and was summarized in a recent review (Hankey and Marshall, 2017). However, other studies have suggested that higher population density is positively associated with higher concentrations of PM_2.5_ (Carozzi and Roth, 2020), but they do not adjust for intersection density and the presence of mass transit infrastructure. In our analyses, higher population density was associated with lower levels of PM_2.5_ before and after adjustment for other factors.

Higher intersection density was associated with higher levels of PM_2.5_. Intersection density combines information about street design and connectivity and is an indicator of walkability; that is, a high intersection density corresponds to a more walkable neighborhood. It could be posited that more walkable areas would have lower air pollution emissions due to lower levels of individual vehicle use. However, studies have shown that levels of PM_2.5_ are higher often in more walkable neighborhoods (Hankey et al., 2012; James et al., 2015) because higher intersection density is associated with lower speed and increased stop and go traffic (Ryan and LeMasters, 2007). Higher intersection density was associated with higher levels of PM_2.5_ before and after adjustment for other factors.

Consistent with our hypothesis, we found that the presence of Bus Rapid Transit systems or subway infrastructure was associated with decreased levels of ambient PM_2.5_ after adjustment for other variables. Evaluated at the mean PM_2.5_ of the sample (12.35 μg/m^3^), we estimate that the presence of this infrastructure is associated with 15% lower PM_2.5_ (95% CI: 3, 27). This is consistent with prior research showing that public transport tends to have an impact on air pollution emissions through a decrease in individual road transport (Ding et al., 2016; Sun et al., 2019). For example, the introduction of a Bus Rapid Transit network in the Mexico City Metropolitan Area led to a significant reduction in the concentration of air pollutants (Bel and Holst, 2018), while the opening of subway stops has been associated with decreases in PM in specific cities (Chen and Whalley, 2012; da Silva et al., 2012). A global study of 43 cities estimated a decrease of 4% in PM around the city center following the opening of a subway system (Gendron-Carrier et al., 2018). Although the value of urban interventions tends to be assessed by their environmental impacts, they are rarely evaluated on their potential health benefits even if these have been estimated to be a significant portion of capital costs (Gendron-Carrier et al., 2018). Urban populations will continue to grow, as will their need for mobility. Improving the movement of populations across urban spaces without significant impact on the environment can only be achieved by expanding mass transit systems.

Motorization rate (# of registered vehicles per 1000 inhabitants) was also an independent predictor of PM_2.5_ in the subsample of 241 cities for which motorization rate was available. Motor vehicle emissions are known to be responsible for a considerable fraction of urban air pollution (Forehead and Huynh, 2018) and efforts are being taken to reduce this source of pollution, especially in countries with rapid urbanization and socioeconomic development (Wu et al., 2017). Interestingly, the association of higher population density with lower PM_2.5_ was weakened when motorization rate was added to the model suggesting, that at least part of the association with population density is due to lower motorization rates in denser areas.

Overall, the associations we observed are consistent with what is known about what are the likely main sources of PM_2.5_ in cities, and Latin America. Research shows that major contributors for the deteriorating air quality in the region are the vehicle fleet, industrial sources and biomass burning (Barraza et al., 2017). Some of the factors we examined in this study correlates directly to transportation sources and although we did not have variables linked to the other major sources, we can speculate that GDP may capture some of them. Given the remarkable growth in motorization across the region over the past decades, and the ability to impact transport options in cities via urban planning and transportation policy a focus on transport related sources is important for future policy decisions.

An important strength of our study is the investigation of the universe of Latin American cities with 100,000 people or more using a wealth of harmonized variables characterizing built environment and other features that could affect PM_2.5_ levels. Cities were defined based on administrative areas and may not coincide with other functional definitions such as metropolitan areas. Because some of the administrative units were large, they included urbanized and less urbanized areas. This might have implications for some of the urban measures we evaluated, such as measures of fragmentation, for which no statistically significant associations were detected.

Our descriptive, cross-sectional analyses cannot be used to prove causality and the intercorrelation between variables makes isolating their independent associations difficult. For these reasons we show associations both before and after adjustment, as both are informative. For example, travel delay showed the highest correlation with population density and was significantly associated with PM_2.5_ in the univariate model, but the association was weaker in adjusted analysis. Greenness and intersection density were both statistically significant in univariate models, but only intersection density remained significant in the multivariate model. Hence, all factors associated with PM_2.5_ whether in unadjusted or adjusted analyses, deserve further research and policy attention as important contributors to city levels of PM_2.5_.

In conclusion, we found that 58% of residents in the Latin American cities examined live in areas that exceed WHO guidelines for PM_2.5_. We also found considerable variation across countries and cities. Informing decision-makers about the quality of the air in cities is an important step towards meeting air quality guidelines that guarantee greater health protection. Furthermore, PM_2.5_ was associated with distinct built environment features including population density, the presence of mass transit infrastructure, intersection density, motorization rate, and GDP per capita. Increasing knowledge about drivers of air pollution in urban areas will help devise interventions towards creation of healthy urban environments that will promote better quality of life for all urban residents.

## CRediT authorship contribution statement

**Nelson Gouveia:** Conceptualization, Writing – original draft, Writing – review & editing. **Josiah L. Kephart:** Formal analysis, Writing – review & editing. **Iryna Dronova:** Writing – review & editing. **Leslie McClure:** Writing – review & editing. **José Tapia Granados:** Writing – review & editing. **Ricardo Morales Betancourt:** Writing – review & editing. **Andrea Cortínez O'Ryan:** Writing – review & editing. **José Luis Texcalac-Sangrador:** Writing – review & editing. **Kevin Martinez-Folgar:** Writing – review & editing. **Daniel Rodriguez:** Conceptualization, Writing – review & editing. **Ana V. Diez-Roux:** Conceptualization, Writing – review & editing.

## Declaration of competing interest

The authors declare that they have no known competing financial interests or personal relationships that could have appeared to influence the work reported in this paper.
